# The Accuracy of Saps Ii and Sofa Score to Predict Mortality in Immunosuppressed Patients Admitted in Intensive Care

**DOI:** 10.1186/2197-425X-3-S1-A526

**Published:** 2015-10-01

**Authors:** PB Medeiros, M Monteiro, T Cardoso, GC Campello

**Affiliations:** Hospital de Santo António, Centro Hospitalar do Porto, Intensive Care Unit, Porto, Portugal

## Introduction

Immunosuppressed patients are a special group in intensive care (ICU), with predisposition to more serious illness. It is essential to accurately predict their clinical outcomes after admission in the ICU.

## Objectives

To study independent risk factors associated with hospital mortality in immunosuppressed patients admitted into the ICU and to evaluate the accuracy of Simplified Acute Physiology Score (SAPS) II and Sequential Organ Failure Assessment (SOFA) scores to predict hospital mortality in this group.

## Methods

Retrospective cohort study conducted in a mixed ICU of a tertiary care, university-affiliated hospital, including all immunosuppressed patients admitted between 2011 and 2014.

The association of the following variables with hospital mortality was studied through logistic regression models: sex, age, type and duration of immunosuppression, infection, microbiological identification, SAPS II and SOFA (24h after admission). Two final models including significant (p < 0.2) and clinically relevant variables were built (one included acute dysfunctions variables and the other did not). the accuracy of the final models and of the physiological scores was calculated using the area under the receiver operating characteristics curve (AU-ROC).

## Results

91 patients were included in the study, aged (mean ± SD) 59 ± 15 years, 54% were male. Median (IQR) length of stay was 7 (2-10) days. Immunosuppression was due to autoimmune disease in 28 (31%) patients, solid tumors in 18 (20%), hematological disease in 14 (15%) and solid organ transplant in 26 (28%). Immunosuppressive treatment was corticosteroids in 61 patients (34%), chemotherapy in 29 (32%) and other medication in 45 (50%); 67 patients (74%) were under more than one immunosuppressive treatment. Mean ± SD SAPS II was 52 ± 21 and SOFA was 9 ± 4. Hospital mortality rate was 48% (n = 44). in the model that included acute dysfunctions, the only independent variable associated with hospital mortality was hematologic dysfunction (SOFA score ≥1) [adjusted OR (95% CI) = 5.06 (1.55-16.46)]. in the model that did not include acute dysfunctions, hospital mortality was only associated with microbiologic documentation of infection [adjusted OR (95% CI) = 3.14 (1.07-9.19)]. the adjusted OR (95% CI) for SAPS was 1.035, per point (1.011-1.060) and for SOFA was 1.220, per point (1.071-1.390). the AU-ROC curve (95% CI) for the first model was 0.58 (0.46-0.70), 0.69 (0.58-0.80) for the second model, 0.68 (0.57-0.79) for SAPS II and 0.70 (0.59-0.81) for SOFA score.

## Conclusions

SAPS II and SOFA did not show good discrimination power to predict hospital mortality in immunosuppressed patients admitted into ICU care. Prospective studies on better discrimination variables are needed for this specific group.
